# CAR-NK Cells Targeting HER1 (EGFR) Show Efficient Anti-Tumor Activity against Head and Neck Squamous Cell Carcinoma (HNSCC)

**DOI:** 10.3390/cancers15123169

**Published:** 2023-06-13

**Authors:** Juliette Nowak, Marco Bentele, Ivana Kutle, Katharina Zimmermann, Jonathan Lukas Lühmann, Doris Steinemann, Stephan Kloess, Ulrike Koehl, Willi Roßberg, Amed Ahmed, Dirk Schaudien, Lavinia Neubert, Jan-Christopher Kamp, Mark P. Kuehnel, Athanasia Warnecke, Axel Schambach, Michael Morgan

**Affiliations:** 1 Institute of Experimental Hematology, Hannover Medical School, 30625 Hannover, Germany; nowak.juliette@mh-hannover.de (J.N.); bentele.marco@mh-hannover.de (M.B.); kutle.ivana@mh-hannover.de (I.K.); zimmermann.katharina@mh-hannover.de (K.Z.); 2 Department of Human Genetics, Hannover Medical School, 30625 Hannover, Germany; luehmann.jonathan@mh-hannover.de (J.L.L.); steinemann.doris@mh-hannover.de (D.S.); 3 Institute for Cellular Therapeutics, Hannover Medical School, 30625 Hannover, Germany; kloess.stephan@mh-hannover.de (S.K.); koehl.ulrike@mh-hannover.de (U.K.); 4 Institute of Clinical Immunology, University Leipzig, 04103 Leipzig, Germany; 5 Fraunhofer Institute for Cell Therapy and Immunology, IZI, 04103 Leipzig, Germany; 6 Department of Otolaryngology, Head and Neck Surgery, Hannover Medical School, 30625 Hannover, Germany; rossberg.willi@mh-hannover.de (W.R.); ahmed.amed@mh-hannover.de (A.A.); warnecke.athanasia@mh-hannover.de (A.W.); 7 Fraunhofer Institute for Toxicology and Experimental Medicine, ITEM, 30625 Hannover, Germany; dirk.schaudien@item.fraunhofer.de; 8 Institute of Pathology, Hannover Medical School, 30625 Hannover, Germany; neubert.lavinia@mh-hannover.de (L.N.); mkuehnel@ukaachen.de (M.P.K.); 9 Biomedical Research in Endstage and Obstructive Lung Disease Hannover (BREATH), German Center for Lung Research (DZL), 30625 Hannover, Germany; kamp.jan-christopher@mh-hannover.de; 10 Department of Respiratory Medicine, Hannover Medical School, 30625 Hannover, Germany; 11 Division of Hematology/Oncology, Boston Children’s Hospital, Harvard Medical School, Boston, MA 02115, USA

**Keywords:** chimeric antigen receptor, CAR-NK cells, NK-92 cells, HER1, EGFR, CD44v6, EpCAM, HNSCC, head and neck cancer, immunotherapy

## Abstract

**Simple Summary:**

Despite new therapeutic approaches in the last decades, prognosis and survival rates for head and neck squamous cell carcinomas (HNSCC) remain poor. Due to its high immune cell infiltration, immunotherapy has become a valuable tool for HNSCC, yet only three monoclonal antibodies have been approved to treat HNSCC. The aim of this study was to develop novel immunotherapeutic strategies for HNSCC employing CAR-NK cells that target HER1/epidermal growth factor receptor (EGFR), which is overexpressed in over 80% of HNSCC patients. We confirmed CAR-NK cell function by assessing cytotoxic killing, IFNγ secretion and CD107a degranulation in 2D and 3D co-culture models. Analyses of primary HNSCC cells that were still viable after anti-HER1 CAR-NK-92 cell challenge showed a high percentage of CD44v6-positive cells, indicating that targeting HER1 alone was not sufficient to eliminate this potential cancer stem cell population, which could contribute to disease progression such as metastasis. We conclude that CAR-NK cell therapy may be a useful tool for HNSCC treatment in combinatorial therapeutic regimens.

**Abstract:**

(1) Background: HNSCC is a highly heterogeneous and relapse-prone form of cancer. We aimed to expand the immunological tool kit against HNSCC by conducting a functional screen to generate chimeric antigen receptor (CAR)-NK-92 cells that target HER1/epidermal growth factor receptor (EGFR). (2) Methods: Selected CAR-NK-92 cell candidates were tested for enhanced reduction of target cells, CD107a expression and IFNγ secretion in different co-culture models. For representative HNSCC models, patient-derived primary HNSCC (pHNSCC) cell lines were generated by employing an EpCAM-sorting approach to eliminate the high percentage of non-malignant cells found. (3) Results: 2D and 3D spheroid co-culture experiments showed that anti-HER1 CAR-NK-92 cells effectively eliminated SCC cell lines and primary HNSCC (pHNSCC) cells. Co-culture of tumor models with anti-HER1 CAR-NK-92 cells led to enhanced degranulation and IFNγ secretion of NK-92 cells and apoptosis of target cells. Furthermore, remaining pHNSCC cells showed upregulated expression of putative cancer stem cell marker CD44v6. (4) Conclusions: These results highlight the promising potential of CAR-NK cell therapy in HNSCC and the likely necessity to target multiple tumor-associated antigens to reduce currently high relapse rates.

## 1. Introduction

Head and neck squamous cell carcinomas (HNSCC) are the sixth most common form of cancer worldwide with 380,000 deaths per year globally and a low 5-year survival rate of 40–50% for human papilloma virus (HPV)-negative patients [1,2]. Risk factors for HNSCC development mainly consist of high alcohol and tobacco consumption and HPV infection. The gold standard treatment options to combat HNSCC include combinations of surgery, chemotherapy and radiation. Within the last decades, monoclonal antibodies have been approved as immunotherapeutic approaches to treat HNSCC. For example, cetuximab targets HER1 (epidermal growth factor receptor, EGFR) and showed superior overall survival in recurrent/metastatic (r/m) HNSCC in combination with platinum-based chemotherapy [3]. The approved anti-PD1 immune checkpoint inhibitors (ICI) pembrolizumab and nivolumab showed improved survival rates compared to conventional therapies for r/m HNSCC [4]. However, due to treatment with ICIs, acute toxicities and chronic immune-related adverse events can affect a high number of the patients and limit therapeutic success.

Alternatively, studies with chimeric antigen receptor (CAR) modified immune cells have recently emerged and present highly promising advances in HNSCC due to improved recognition of cancer cells [5,6,7,8]. Moreover, a Phase I clinical trial testing a T4-CAR T cell approach targeting the ErbB family demonstrated safe intratumoral administration and stable disease with few adverse effects [9], and additional clinical trials in HNSCC using CAR-T cells targeting MUC1, PD-1, EpCAM and EBV are currently recruiting (NCT05239143, NCT05117138, NCT03740256, NCT05639972, NCT02915445, NCT04847466). While CAR-T cell therapy has shown tremendous success in B cell malignancies, effects in solid tumors have faced multiple challenges, including immunosuppressive tumor microenvironments (TME), choice of target antigens due to intra- and inter-patient tumor heterogeneity, as well as T cell homing and persistence [10]. Furthermore, severe side effects of CAR-T cell therapy were observed in the early clinical trials, including cytokine release syndrome (CRS) and neurotoxicity, which can be lethal [11].

To overcome these challenges, we chose to exploit the natural ability of NK cells to directly kill target cells and simultaneously trigger an adaptive immune response. Previous studies showed that CAR-NK cells were able to effectively kill CD19^+^ leukemic cells [12]. Furthermore, NK cells harbor a high potential for an off-the-shelf product, based on the lack of need for HLA matching and use of allogenic sources without severe adverse effects [13]. Additionally, irradiated NK-92 cells can be administered in high doses to patients and have shown strong safety track records [14]. The possibility to use NK-92 cell lines for clinical applications greatly decreases the costs for off-the-shelf CAR treatments, which supports further CAR-NK-92 cell research. Here, we investigated CAR-NK-92 cells designed to target HER1, which is overexpressed in over 80% of HNSCC [15]. Furthermore, HER1 expression is responsible for tumor proliferation, formation of metastasis and treatment resistance, and hence associated with poor prognosis, making it a compelling target antigen [16].

In the present study, we compared cytotoxicity of six anti-HER1 CAR-NK-92 cell variants, generated primary HNSCC (pHNSCC) cell lines and demonstrated effective elimination of pHNSCC cells as well as SCC cell lines by our anti-HER1 CAR-NK-92 cells in 2D and 3D models. Anti-HER1 CAR-NK-92 cells also displayed increased apoptosis induction, CD107a expression and IFNγ secretion in co-culture with target cells, emphasizing the promising potential of CAR-NK cell therapy in HNSCC.

## 2. Materials and Methods

### 2.1. Cell Culture and Cell Lines

SCC-4 and SCC-25 cell lines (DSMZ, Braunschweig, Germany) were cultivated in DMEM/F12 GlutaMax medium (Thermo Fisher Scientific, Waltham, MA, USA) supplemented with 10% fetal bovine serum (FBS Standard, PAN-Biotec, Aidenbach, Germany) and 1% penicillin/streptomycin (P/S; PAN-Biotec). NK-92 cells (DSMZ) were cultivated in RPMI 1640 medium (PAN-Biotec) supplemented with 10% FBS, 1% P/S, 1 mM sodium pyruvate (PAN-Biotec) and 400 U/mL human IL2 (PeproTech, Winterhude, Germany). All cells were cultured under standard humidified conditions at 37 °C and 5% CO_2_.

### 2.2. Isolation and MACS-Sorting of Primary HNSCC Cells

Primary HNSCC (pHNSCC) tumor samples were obtained from the Department of Otolaryngology at Hannover Medical School following patient informed consent according to the Declaration of Helsinki and following approval from the local ethics committee (No. 9573_BO_K2021). pHNSCC tumor pieces were digested in 1× HBSS (GIBCO, Life Technologies, Carlsbad, CA, USA) containing 3 mg hyaluronidase (Sigma–Aldrich, St. Louis, MO, USA) and 10 mg collagenase II (StemCell Technologies, Vancouver, BC, Canada). Erythrocytes were lysed using lysis buffer (BD Biosciences, Franklin Lakes, NJ, USA). Isolated pHNSCC were immediately stored at −180 °C, analyzed or MACS sorted. pHNSCC were MACS-sorted for EpCAM according to the manufacturer’s instructions (Miltenyi Biotec, Bergisch Gladbach, Germany). Sorted cells were cultured in PneumaCult Ex medium (StemCell Technologies) supplemented with 50× supplement and 1% P/S at 37 °C and 5% CO_2_.

### 2.3. Generation of Retroviral Vectors and Viral Supernatant Production

Third generation CARs carrying CD28, 41BB and CD3ζ domains were cloned into self-inactivating alpharetroviral backbones carrying an IRES-eGFP cassette for easy detection [17]. Three different anti-HER1 single-chain variable-fragments (scFv) derived from different patents were used: **7D6**, **12D3** (WO 2Ull/156ol7 A2) and **2F8** (WO 02/100348 A2). Alphraretroviral vectors were pseudotyped with RD114TR, while lentiviral vectors were pseudotyped with VSVg. All viral vector supernatants were generated using HEK-293T cells (DSMZ) and calcium-phosphate transfection with split packaging design (alpharetroviral vector production: 5 μg alpharetroviral vector construct, 2.5 μg pcDNA3-α-gag/pol.co and 2 μg phCMV-RD114TR; lentiviral vector production: 5 μg lentiviral vector construct, 12 μg pcDNA3.GP.4xCTE, 6 μg pRSV-Rev and 2 μg pMD.G-VSVg). 

### 2.4. Transduction of Cell Lines with Retroviral Vectors

Transduction was done on retronectin (Takara Bio, Otsu, Japan) coated 48-well plates. Wells were loaded with viral supernatant and centrifuged (400× *g*, 4 °C, 30 min). Next, 1 × 10^5^ NK-92 cells were seeded per well. For transduction with the lentiviral vector, 1 × 10^5^ SCC or pHNSCC cells were seeded on a 24-well plate. Next, viral supernatant was added in medium containing protamine sulfate. Transduced NK-92 cells and all target cells were sorted at the cell sorting core facility at Hannover Medical School.

### 2.5. Bradford Assay and Western Blotting

Total protein concentrations of cell lysates were determined with the Coomassie dye-binding assay (Bio-Rad Laboratories, Hercules, CA, USA). For Western blot analysis, membranes were incubated with anti-CD3ζ-HRP (1:1000, Santa Cruz Biotechnology, CA, USA) in 5% milk. After detection, membranes were stripped and reprobed with anti-GAPDH-HRP (1:10,000, GeneTex/BIOZOL, Eching, Germany) in 5% milk.

### 2.6. gDNA Isolation and Vector Copy Number Determination

To determine vector copy number, gDNA was isolated using the QIAamp DNA blood Mini Kit (QIAGEN, Hilden, Germany) and amplified using Taqman qPCR technology using Taqman Fast Advanced Master Mix according to manufactures instruction (Thermo Fisher Scientific). (Sequences: *WPRE* forward GAGGAGTTGTGGCCCGTTGT; *WPRE* reverse TGACAGGTGGTGGCAATGCC; *WPRE* probe CTGTGTTTGCTGACGCAAC; *PTBP2* forward TCTCCATTCCCTATGTTCATGC; *PTBP2* reverse GTTCCCGCAGAATGGTGAGGTG; *PTBP2* probe ATGTTCCTCGGACCAACTTG).

### 2.7. Optical Genome Mapping

Optical Genome Mapping (OGM) was performed according to the manufacturer’s instructions. Briefly, DNA was isolated from 1.5 × 10^6^ frozen cells using the Bionano Prep SP Frozen Cell Pellet DNA Isolation Protocol v2 (#30398 Rev B, Bionano Genomics Inc., San Diego, CA, USA). The DNA concentration was determined with the Qubit^TM^ dsDNA BR Assay Kit and a Qubit 3.0 Fluorometer (Thermo Fisher Scientific). Subsequently, the DNA was labeled with Bionano Prep Direct Label and Stain Kit (Protocol #30206 Rev G; Bionano Genomics Inc.) and the concentration determined with the Qubit^TM^ dsDNA HS Assay Kit and a Qubit 3.0 Fluorometer (Thermo Fisher Scientific). The labeled DNA was loaded on a Saphyr G2.3 chip and imaged with the Saphyr device (Bionano Genomics Inc.). The data were analyzed and visualized on the Bionano Access Server with the de novo pipeline (Tools Version 1.7.1, reference genome GRCh38/hg38). The recommended filter settings were applied and the frequency of SVs in the Bionano control database was set to 0%. 

### 2.8. Flow Cytometry: Cytotoxicity Assay

For detection, target cells were stained with 1 μM FarRed CellTrace according to manufacturer’s instruction (Thermo Fisher Scientific) and then seeded in RPMI medium on one 48-well plate/per time point (0, 24, 48 h). The next day, unmodified and CAR-NK-92 cells were seeded on top in specific effector:target cell (E:T) ratios. For each time point, co-cultures were stained with anti-HER1 BV605 (BioLegend, San Diego, CA, USA) and/or anti-CD44v6 PE (R&D Systems, Minneapolis, MN, USA) and analyzed with a CytoFLEX S flow cytometer (Beckman Coulter, Brea, CA, USA) to acquire events in 80 μL. Cytotoxicity was calculated as shown below, whereby the proliferation rate of target and effector cells was factored in.

$$\mathrm{remaining}\;\mathrm{target}\;\mathrm{cells}=\frac{\mathrm{target}\;\mathrm{cells}\;}{\left(\mathrm{target}\;\mathrm{cells}\;+\;\mathrm{effector}\;\mathrm{cells}\right)}*100$$

### 2.9. Automated Live-Cell Imaging: Cytotoxicity Assay and 3D Spheroid Assay

For cytotoxicity assays, 0.35–1.2 × 10^4^ mCherry^+^ target cells were seeded on 96-well plates. The next day, CAR-NK-92 cells were seeded in complete RPMI medium containing IL-2, 1 mM CaCl_2_ and Annexin V NIR dye (Sartorius, Göttingen, Germany). For spheroid formation, 0.35–1.5 × 10^4^ mCherry^+^ SCC-4, SCC-25 or primary HNSCC cells were seeded on ultra-low attachment 96-well plates (S-BIO, Singapore, Central Region, Singapore). After 72 h, CAR-NK-92 cells were seeded on top. Plates were analyzed using Incucyte SX5 Live-Cell Analysis instrument (Sartorius).

### 2.10. CD107a Degranulation Assay

NK-92 cells were starved of IL-2 for 24 h before and throughout the duration of the experiment. The next day, NK-92 cells were seeded together with target cells using an E:T ratio of 1:1 in complete RPMI medium. Co-cultures were incubated with monensin, Brefeldin A and anti-CD107a antibody (Miltenyi Biotec) and cultivated for 4 h total. Next, cells were incubated with anti-Flag PE (BioLegend, San Diego, CA, USA) and anti-CD56 PC7 (BD Biosciences, Franklin Lakes, NJ, USA). Afterwards, the cells were fixed with fixation buffer (BioLegend) and analyzed using a CytoFLEX S (Beckman Coulter).

### 2.11. IFNγ Secretion 

NK-92 cells were starved of human IL-2 for 24 h prior to seeding and during the co-culture experiment (except for the screening of six anti-HER1 CAR-NK-92 cells, which was conducted with human IL-2). IFNγ secretion was analyzed using human IFNγ ELISA according to the manufacturer’s instructions (BioLegend). Briefly, the IFNγ standards provided by the ELISA kit were used as positive controls (7.8, 15.6, 31.3, 62.5, 125, 250 and 500 pg/mL) and 1X Assay Diluent A was used as the zero standard (0 pg/mL) or negative control. Dilutions of the IFNγ standard were used to quantify IFNγ released from the NK-92 cells according to the manufacturer´s instructions. 

### 2.12. Immunohistochemistry Staining and Analysis

Immunohistochemical staining was performed on paraffin sections (2 μm thickness). All sections were deparaffinized with xylene twice, rehydrated using decreasing ethanol concentrations, and then subjected to heat-induced epitope retrieval in antibody buffer. Stainings were performed using the antibodies listed in Supplementary Table S1, the ZytoChem Plus HRP Polymer Kit (Zytomed Systems, Berlin, Germany), 3,3′-diaminobenzidine solution (DAB), hematoxylin counterstain and the Eukitt mounting medium. Stained slides were digitized using the Nano Zoomer S210 (Hamamatsu Photonics, Herrsching a. Ammersee, Germany). DAB-positive and negative cells were counted with the nuclei detection image analysis application of Visiopharm (Visiopharm, Hørsholm, Denmark).

### 2.13. Statistical Analysis

Statistical analysis was performed via GraphPad Prism 6.01 software (GraphPad Software, San Diego, CA, USA) and two-way ANOVA with Bonferroni’s post-hoc test. * indicates comparison to unmodified NK-92 cells within time points (e.g., 24 h to 24 h) unless specified otherwise. Error bars in all figures depict the standard deviation of the mean. * = *p* < 0.05, ** = *p* < 0.01, *** = *p* < 0.001, **** = *p* < 0.0001.

## 3. Results

### 3.1. Validation of Anti-HER1 CAR Expression in NK-92 Cells

**Figure 1:**
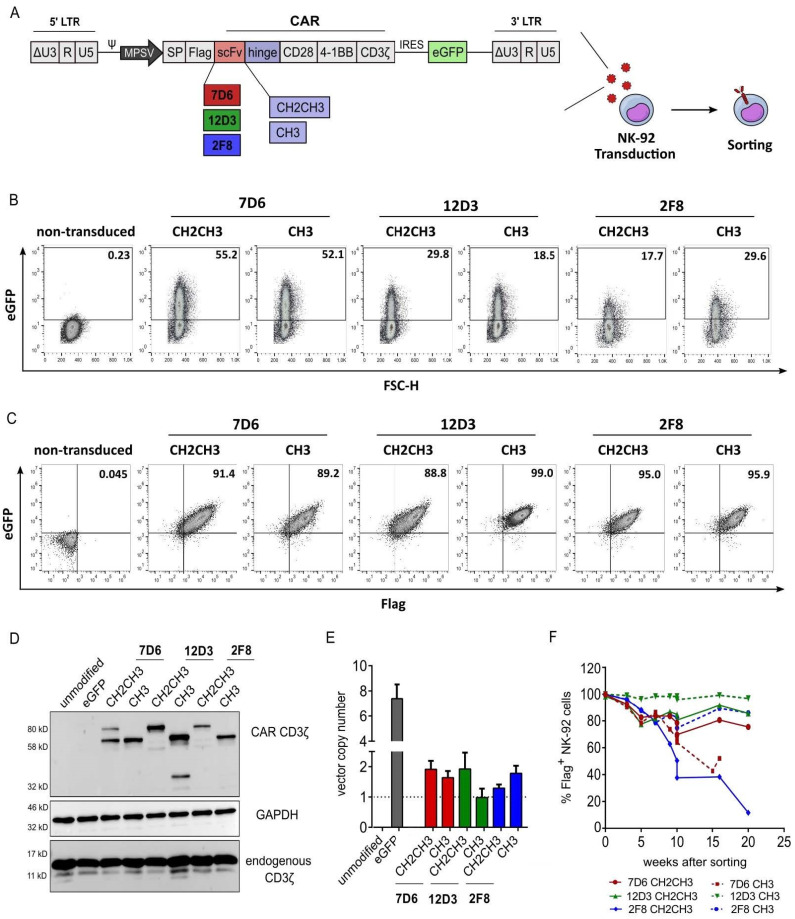
Transduction and expression of six anti-HER1 CAR-NK-92 candidates. (**A**) Vector design showing self-inactivating alpharetroviral backbones, third generation CAR and an IRES-eGFP cassette. Three anti-HER1 single chain variable fragments (scFv) 7D6, 12D3 and 2F8 were each combined with long (CH2CH3) and medium (CH3) hinges to generate six anti-HER1 CAR-NK-92 candidates. (**B**) Transduction efficiency was assessed by percentage of eGFP^+^ expression via flow cytometry. (**C**) CAR expression 3-weeks post sorting for flag-APC^+^eGFP^+^ cells. Analysis of CAR expression and vector integration via (**D**) Western blot (CAR CD3ζ = 60 or 80 kDa, GAPDH = 37–38 kDa, endogenous CD3ζ = 15–18 kDa), see the Supplementary File S1 for the original images of Western blot. (**E**) vector copy number analysis and (**F**) flow cytometry. Mean with standard deviation shown, n = 3.

Six CAR-NK variants were created using single-chain variable fragments (scFv) 7D6, 12D3, 2F8, and each of which was combined with a long (CH2CH3) and a medium (CH3) hinge version (Figure 1A). Transduction efficiency was assessed using eGFP expression six days post-transduction (Figure 1B). To generate high-purity CAR-NK-92 cells, cells were sorted for flag-APC^+^eGFP^+^ expression and demonstrated >88% flag-tag, hence CAR surface expression, three weeks after sorting (Figure 1C). Western blot analysis was used to validate full-length CAR-CD3ζ protein expression, which varied in size (60 or 80 kDa) depending on the IgG1-based hinge type used (long hinge: CH2CH3 vs medium hinge: CH3). The 12D3 CH3 CAR variant showed an additional protein band at 40 kDa, which may be a degradation product. Unmodified and eGFP-transduced NK-92 cells serve as controls, which lack CAR-based CD3ζ expression and only showed endogenous CD3ζ expression at 15–18 kDa, which was also apparent for all transduced CAR variants (Figure 1D). Determination of vector copy number (VCN) quantifies the *wPRE* sequence of the alpharetroviral vector backbone in relation to *PTBP2* as a housekeeping control. Similar vector integrations levels were achieved in all CAR-NK-92 cells. eGFP-NK-92 control cells (no CAR) showed VCN of 7.41, indicating a better transduction efficiency, possibly due to the smaller vector size (Figure 1E). CAR-NK-92 cells were monitored for flag-tag expression via flow cytometry over several weeks after sorting and a loss of CAR expression was observed for variants 7D6 CH3 (<60%) and 2F8 CH2CH3 (<20%) after 15–20 weeks. All remaining variants maintained a stable CAR expression as indicated by the expression of the flag-tag in more than 80% of cells over several weeks (Figure 1F).

### 3.2. Functional Screening of Six Anti-HER1 CAR-NK-92 Cell Variants

**Figure 2:**
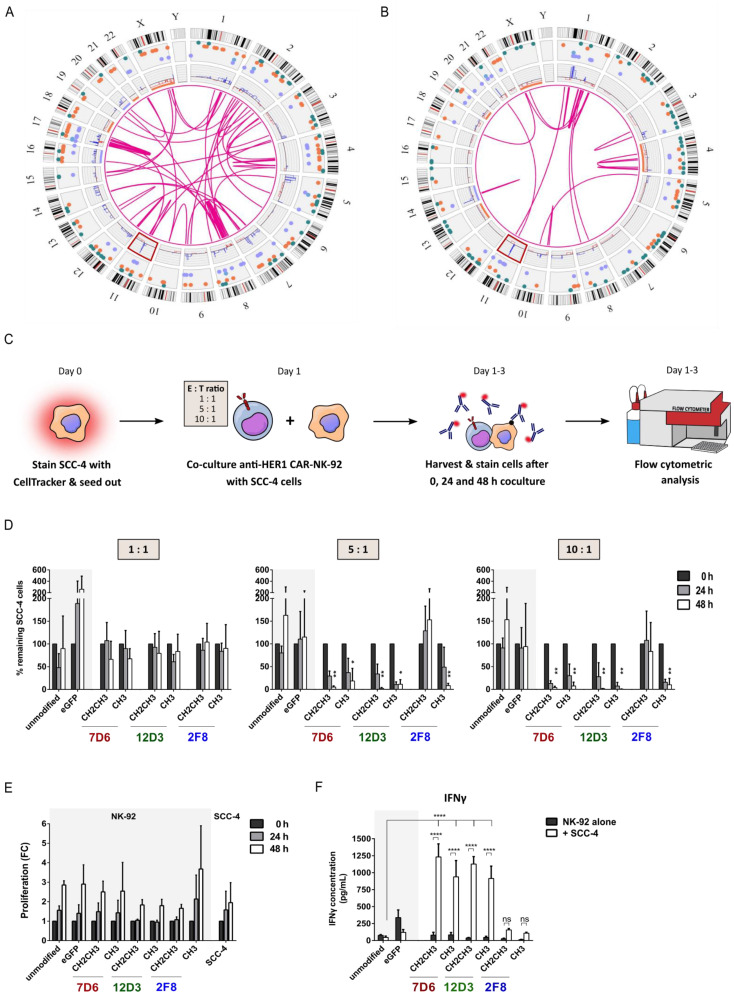
Functional screening of six anti-HER1 CAR-NK-92 cell candidates. Characterization of SCC-4 cells (**A**) and SCC-25 cells (**B**) using OGM. Red box highlights amplification at 11q13 (Legend: ● copy number gains (blue lines), ● copy number losses (red lines), ● insertions, ● deletions, ● inversions, ● duplications, ● intra-fusions or inter-translocations (pink lines)). (**C**) Experimental design of flow cytometry-based cytotoxicity assay. (**D**) Cytotoxicity assay using anti-HER1 CAR-NK-92 candidates and SCC-4 cells. (**E**) Proliferation of CAR-NK-92 cells in co-cultures and SCC-4 cell monocultures during cytotoxicity assay. (**F**) IFNγ ELISA using supernatant from 48 h time point of cytotoxicity assay (5:1 E:T ratio). E:T = effector:target cell ratio. Mean with standard deviation shown. Statistical analysis was performed using two-way ANOVA with Bonferroni’s post-hoc test for comparison to unmodified NK-92 cells within the same time points (e.g., 24 h to 24 h), n = 3. * = *p* < 0.05, ** = *p* < 0.01, **** = *p* < 0.0001.

Prior to assessing cytotoxic functionality, we characterized two HNSCC cell lines SCC-4 and SCC-25 using Optical Genome Mapping (OGM). Both cell lines clearly depicted the genetic heterogeneity of this cancer as evidenced by the different types of genetic aberrations, including aneuploidies, translocations, duplications and deletions. While SCC-4 cells (Figure 2A) showed a more complex profile of genetic aberrations compared to SCC-25 cells (Figure 2B), some similarities were detected, including high-fold amplification on chromosome 11q13 (Figure 2, Supplementary Figure S1). This region harbors genes associated with tumor progression and cell cycle control, including cyclin D1/PRAD1 and BCL-1 [18]. To ensure recognition by the various anti-HER1 CAR-NK-92 cells, SCC-4 and SCC-25 cells were examined for HER1 expression and found to be highly positive (>99%) (Supplementary Figure S1). For the functional screening, the genetically more complex cell line SCC-4 was used as a target cell line. Here, SCC-4 cells were harvested, stained with FarRed CellTrace and seeded on day 0. The next day, co-cultures of all six anti-HER1 CAR-NK-92 variants were seeded using different E:T ratios. Cells were harvested at 0, 24 and 48 h after initiation of co-culture, stained for HER1 and analyzed using flow cytometry (Figure 2C). At 5:1 and 10:1 E:T ratios, improved killing effects were observed for all CAR variants after 24 h and 48 h, except for 2F8 CH2CH3 CAR-NK-92 cells. This can be explained by loss of CAR expression seen in Figure 1, as the functionality was assessed around a similar time as the loss of CAR expression was observed. No CAR construct appeared superior to the others regarding killing activity throughout 24 h and 48 h and different E:T ratios (Figure 2D). Furthermore, proliferation rates of 2-3-fold of transduced NK-92 cells were comparable in co-culture compared to unmodified NK-92 cells after 48 h. SCC-4 cells alone also proliferated well, despite the medium change to RPMI with up to 2-fold within 48 h (Figure 2E). As an additional functional readout, IFNγ secretion after 48 h co-culture was assessed via ELISA. Unmodified and eGFP-NK-92 cells showed no increased IFNγ secretion upon co-culture with SCC-4 cells, while all CAR-NK-92 cells showed increased IFNγ secretion in the presence of SCC-4 cells (Figure 2F, IFNγ ELISA of E:T ratios 1:1 and 10:1 found in Supplementary Figure S2).

### 3.3. Anti-HER1 CAR-NK-92 Variants Showed Enhanced Killing, Apoptosis Induction and Degranulation against SCC-4 and SCC-25 Cell Lines

**Figure 3:**
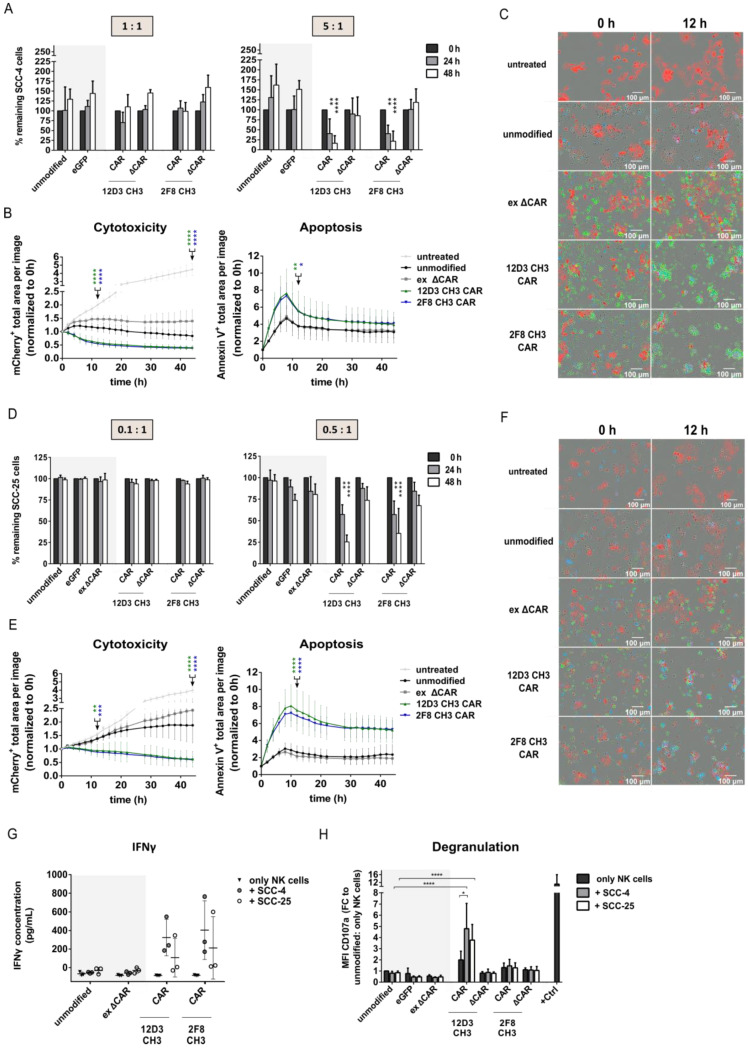
Enhanced cytotoxic capacity, apoptosis induction, IFNγ secretion and degranulation of 12D3 CH3 and 2F8 CH3 CAR-NK-92 cells. (**A**) Flow cytometry-based and (**B**) Incucyte-based cytotoxicity assays against SCC-4 cells. (**C**) Automated live-cell imaging using Incucyte against SCC-4 cells (red: mCherry^+^ SCC-4 cells; green: eGFP^+^ CAR NK-92 cells; blue: Annexin V^+^ cells). (**D**) Flow cytometry-based and (**E**) Incucyte-based cytotoxicity assay against SCC-25 cells. (**F**) Automated live-cell imaging using Incucyte against SCC-25 cells (red: mCherry^+^ SCC-25 cells; green: eGFP^+^ CAR NK-92 cells; blue: Annexin V^+^ cells). (**G**) IFNγ ELISA of 48 h supernatant from co-cultures with SCC-4 cells (5:1 E:T ratio) and with SCC-25 cells (0.5:1 E:T ratio). (**H**) CD107a expression of NK-92 cells after 4 h co-culture of 1:1 E:T ratio for both target cell lines. +Ctrl consists of PMA/Ionomycin stimulated NK-92 cells. E:T = effector:target cell ratio; FC = fold change. Mean with standard deviation shown. Statistical analysis was performed using two-way ANOVA with Bonferroni’s post-hoc test for comparison (**A**,**D**) to unmodified NK-92 cells within time points (e.g., 24 h to 24 h) or (**B**,**E**) between full-length CAR-NK-92 cells and unmodified NK-92 cells at 12 h and 44 h. n = 3, except for Incucyte data n = 9 (Incucyte experiments and ELISA were performed with three separate transduction rounds of NK-92 cells). * = *p* < 0.05, ** = *p* < 0.01, *** = *p* < 0.001, **** = *p* < 0.0001.

Based on stable long-term CAR expression, lack of tonic signaling (Supplementary Figure S3) and HNSCC killing activity, anti-HER1 CAR-NK-92 cell variants 12D3 CH3 and 2F8 CH3 were selected as the most promising candidates and used for all further experiments. For more suitable controls, internally truncated versions of these two CARs that lack signaling and effector domains (ΔCAR) and one externally truncated CAR (ex ΔCAR), that lacks the extracellular scFv and hinge domains were generated and used to transduce NK-92 cells. Flow cytometry-based cytotoxicity assays showed enhanced killing of SCC-4 cells only upon co-culture with full-length 12D3 CH3 and 2F8 CH3 CAR-NK-92 cells after 24 h and 48 h using 5:1 E:T ratios (Figure 3A). This was confirmed using an Incucyte-based cytotoxicity assay, which showed that the strongest killing effect of 12D3 CH3 and 2F8 CH3 CAR-NK-92 cells occurred within 10 h of co-culture initiation. Monocultures of SCC-4 cells continued to proliferate, while co-culture with unmodified NK-92 cells and ex ΔCAR-modified NK-92 cells prevented SCC-4 proliferation, further confirming the flow cytometry-based results. Additionally, Annexin V staining showed enhanced apoptosis rate in co-cultures with both full length CAR-NK-92 cell variants within 10 h of co-culture initiation, which reflected the rapid decline of SCC-4 cells in these co-cultures (Figure 3B). The nearly complete loss of SCC-4 cells upon co-culture with NK-92 cells modified with full-length CARs was also evident in microscopy pictures at 12 h, which indicated clustering of eGFP^+^ CAR-NK-92 cells around the few remaining mCherry^+^ SCC-4 cells compared to co-cultures with unmodified or ex ΔCAR-modified NK-92 cells (Figure 3C). SCC-25 cells were already effectively killed at an E:T ratio of 0.5:1 using 12D3 CH3 and 2F8 CH3 CAR-NK-92 cells after 24 h and 48 h compared to the controls in the flow cytometry-based cytotoxicity assay (Figure 3D). Similar results were obtained with the Incucyte-based cytotoxicity assay. Furthermore, the rate of apoptosis in co-cultures of SCC-25 cells with full-length CAR-NK-92 cells was greatly enhanced within 10 h compared to unmodified and ex ΔCAR-NK-92 control cells, which concurs with data from SCC-4 cells (Figure 3E). During the automated live-cell imaging, the same phenomenon of strong reduction of mCherry^+^ SCC-25 cells was apparent after 12 h in co-cultures with NK-92 cells modified with full-length 12D3 CH3 and 2F8 CH3 CARs. Moreover, greater clustering around SCC-25 cells and higher rates of apoptosis were visible upon co-culture with full-length CAR-NK-92 cells compared to untreated SCC-25 cells or co-cultures with unmodified or ex ΔCAR-NK-92 cells (Figure 3F). As an additional indication of targeted NK-92 cell activity, elevated IFNγ levels were only observed in ELISA analysis after co-culture of SCC-4 or SCC-25 cells with 12D3 CH3 and 2F8 CH3 CAR-NK-92 cells (Figure 3G). The IFNγ secretion of CAR-NK-92 cells in monocultures remained at the same level as unmodified NK-92 cells. To determine the mechanism of cytotoxic killing, we examined expression of degranulation marker CD107a on NK-92 cells after 4 h co-culture with SCC-4 and SCC-25 cells. Monocultures of 12D3 CH3 CAR-NK-92 cells showed a 2-fold upregulation of CD107a MFI compared to unmodified NK-92 cells alone. Moreover, 12D3 CH3 CAR-NK-92 cells also showed an upregulation of CD107a MFI by 5-fold in the presence of SCC-4 cells and by 4-fold in the presence of SCC-25 cells (Figure 3H). 2F8 CAR-NK-92 cells showed no enhanced expression of CD107a, which suggests that target cell killing is likely to occur via another mechanism. A possible mechanism could be induction of death receptor-mediated apoptosis via upregulated expression of TRAIL or FAS ligands; however, this was not assessed in this study.

### 3.4. Isolation and Characterization of Primary HNSCC Tumor-Derived Cells

**Figure 4:**
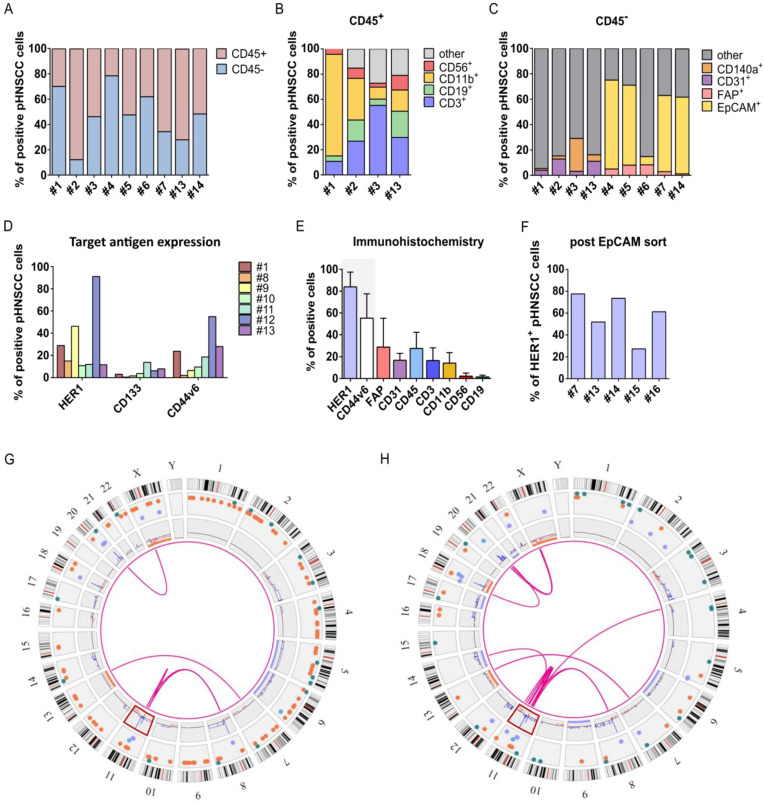
Characterization of primary HNSCC (pHNSCC) cells. Characterization of (**A**) CD45^+^ immune cells present in isolated pHNSCC samples as well as (**B**) percentage of NK cells (CD56^+^), macrophages (CD11b^+^), B cells (CD19^+^) and T cells (CD3^+^) of CD45^+^ cells in pHNSCC samples and (**C**) percentages of stroma cells (CD140a^+^), endothelial cells (CD31^+^), cancer-associated fibroblasts (FAP^+^) and epithelial marker-positive cells (EpCAM^+^) in the CD45^−^ pHNSCC cell population within 24 h from isolation (or thawing). (**D**) Target antigen expression within 24 h of isolation (or thawing) of pHNSCC samples. (**E**) Quantitative analysis of immunohistochemical stainings of seven patient samples (photos from exemplary patient samples shown in Supplementary Figures S6 and S7). (**F**) HER1 expression on EpCAM^+^ sorted and expanded pHNSCC samples. Optical genome mapping of EpCAM^+^ sorted and expanded pHNSCC samples (**G**) #14 and (**H**) #16. Red box highlights amplification of 11q13, (Legend: ● copy number gains (blue lines), ● copy number losses (red lines), ● insertions, ● deletions, ● inversions, ● duplications, ● intra-fusions or inter-translocations (pink lines)). Mean with standard deviation shown. n = 4–9 pHNSCC samples as indicated (**A**–**E**).

To examine effectivity of selected anti-HER1 CAR-NK-92 cells against primary HNSCC (pHNSCC)-derived cells, we isolated and characterized pHNSCC cells (Table 1). Most patient samples were HPV^−^ and of primary occurrence, without treatment at the time of biopsy. To determine the isolated cell types, we assessed expression of hematological markers, stroma, endothelial, cancer-associated fibroblast and epithelial markers. CD45 expression varied highly between patient samples from 21.6% to over 87.5% CD45^+^ cells, suggesting a high presence of immune cells within the tumors (Figure 4A). The CD45^+^ cell population largely consisted of T cells and macrophages, but also contained NK cells, B cells and other cells (Figure 4B). The CD45^−^ population was screened for endothelial cells and stroma cells, which were only present at low percentages (<30%). As EpCAM expression was shown to be highly specific in carcinomas, especially HNSCC [19], we used the epithelial marker EpCAM to characterize the largely unknown cell population in patient samples #1, #2, #3 and #13. Approximately 60–70% of CD45^−^ cells in freshly isolated patient samples were EpCAM^+^, with the exception of sample #6 (Figure 4C). After confirming that SCC-4 and SCC-25 cell lines are also entirely EpCAM^+^ (Supplementary Figure S4), we used a previously published EpCAM^+^ cell sorting approach to establish pHNSCC cell cultures, and used PneumaCult Ex medium (StemCell Technologies, Vancouver, BC, Canada) for improved epithelial growth [20]. However, to get an unbiased idea of target antigen expression, we assessed surface marker expression on freshly isolated pHNSCC samples before sorting. Despite strong variation among samples, HER1 expression was highest, followed by CD44v6, while CD133 was expressed at low very low levels, suggesting HER1 as a promising target antigen (Figure 4D). This was also consistent with immunohistochemical (IHC) staining, which also demonstrated co-localization of HER1^+^ and CD44v6^+^ cells (Supplementary Figures S6 and S7) and stronger HER1 expression than CD44v6. Furthermore, IHC staining also supported a greater presence of CD3^+^ T cells and CD11b^+^ macrophages than CD56^+^ NK cells and CD19^+^ B cells (Figure 4E, Supplementary Figures S6 and S7). Next, we ensured HER1 expression persisted throughout the sorting procedure. HER1 expression exceeded 50% on four out of five patient samples (Figure 4F). Furthermore, HER1 expression on cells from patient sample #7 increased throughout the sorting process from 11.7% to 51.8%, which can be explained by the loss of non-epithelial cells. Expanded EpCAM-sorted pHNSCC samples #14 and #16 were also used for OGM (Figure 4G,H, respectively). Although fewer overall genetic aberrations were observed in the pHNSCC samples as compared to SCC-4 and SCC-25 cell lines, it may be of interest that both patient samples analyzed exhibited the same chromosome 11q13 amplification that was identified in the SCC-4 and SCC-25 cell lines (Figure 4G,H, Supplementary Figure S4).

### 3.5. Anti-HER1 CAR-NK-92 Cells Show Enhanced Killing, Apoptosis and Degranulation against pHNSCC Cells

**Figure 5:**
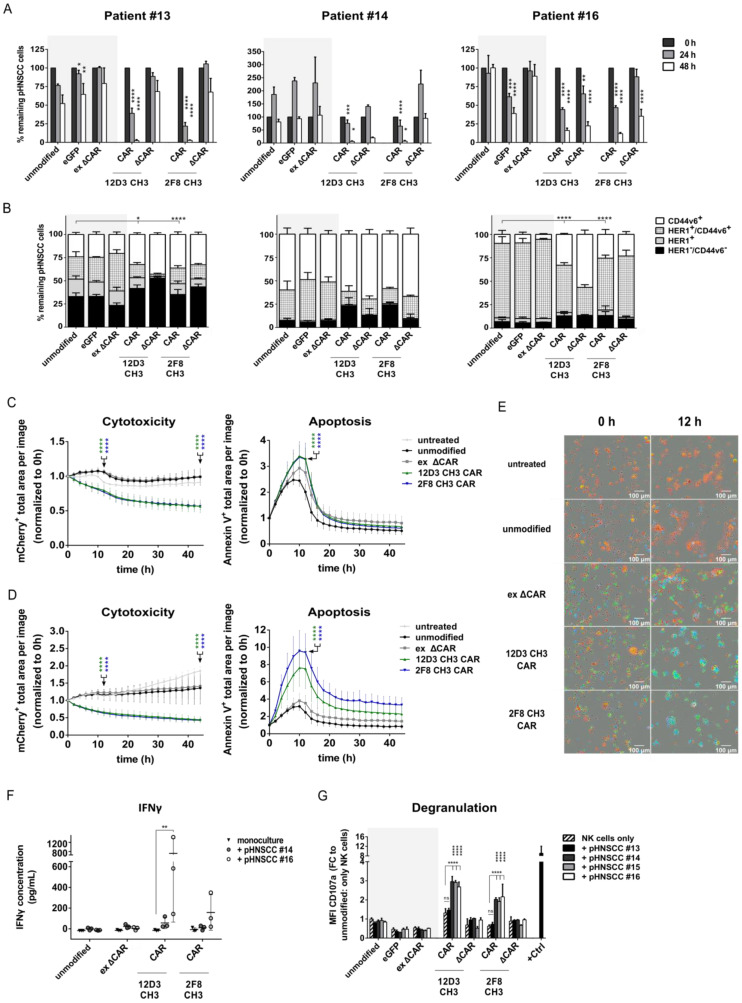
Anti-HER1 CAR-NK-92 cells effectively kill pHNSCC cells. (**A**) Flow cytometry-based cytotoxicity assay against EpCAM^+^ sorted and expanded pHNSCC samples (0.5:1 E:T ratio). (**B**) Target antigen expression profile of remaining pHNSCC after 24 h of co-culture with control NK-92 cells and CAR NK-92 cells. Incucyte-based cytotoxicity assay targeting (**C**) pHNSCC #14 and (**D**) pHNSCC #16 cells (0.5:1 E:T ratio). (**E**) Automated live-cell imaging using Incucyte to monitor NK-92 cell activity against pHNSCC #16 cells (red: mCherry^+^ pHNSCC #16 cells; green: eGFP^+^ CAR NK-92 cells; blue: Annexin V^+^ cells). (**F**) IFNγ ELISA of 48 h supernatant from co-cultures (0.5:1 E:T ratio). (**G**) CD107a expression on NK-92 cells after 4 h co-culture at 1:1 E:T ratio for all target cell lines. +Ctrl consists of PMA/Ionomycin stimulated NK-92 cells. E:T = effector:target cell ratio; FC = fold change. Mean with standard deviation shown. Statistical analysis was performed using two-way ANOVA with Bonferroni’s post-hoc test for comparison (**A**) to unmodified NK-92 control cells within the same time points (e.g., 24 h to 24 h), (**B**) between CD44v6^+^ population of full lengths CAR-NK-92 cells and unmodified NK-92 cells or (**C**,**D**) between full-length CAR-NK-92 cells and unmodified NK-92 cells at 12 h and 44 h. n = 3, except for Incucyte data n = 9 (Incucyte experiments and ELISA were performed with three separate transduction rounds of NK-92 cells). * = *p* < 0.05, ** = *p* < 0.01, *** = *p* < 0.001, **** = *p* < 0.0001.

Enhanced killing of anti-HER1 CAR-NK-92 cells was validated against EpCAM^+^ pHNSCC cell lines in flow cytometry-based cytotoxicity assays using an E:T ratio of 0.5:1. Similarly to previous cytotoxicity assays, significance of killing effects was only statistically analyzed in comparison to unmodified NK-92 cells within the same time points (e.g., 24 h to 24 h). While unmodified NK-92 cells showed a strong cytotoxic killing effect against pHNSCC #13 cells after 48 h, full-length CAR-NK-92 cells had an even stronger cytotoxic effect after 24 h and 48 h. Surprisingly, eGFP-NK-92 control cells demonstrated significantly worse killing of pHNSCC #13 cells compared to unmodified NK-92 cells after 24 h and 48 h (Figure 5A, Patient #13). Unmodified, eGFP and ex ΔCAR control NK-92 cell co-cultures led to 150% of patient #14 cells after 24 h, which indicates an outgrowth of target cells compared to number of target cells seeded at 0 h (100%). This may be due to a naturally faster proliferation rate compared to other pHNSCC cell lines. After 48 h, unmodified, eGFP and ex ΔCAR control NK-92 cells were able to reduce target cells back to a similar number as at 0 h. NK-92 cells modified with 12D3 CH3 and 2F8 CH3 CARs eliminated basically all pHNSCC #14 cells after 48 h co-culture, which is consistent with the elimination of patient #13 cells (Figure 5A, Patient #14). Unexpectedly, internally truncated 12D3 CH3 control NK-92 cells also showed an improved killing effect after 48 h. A similar effect was observed for patient #16, where eGFP and both internally truncated CAR control NK-92 cells also displayed a significant killing effect compared to unmodified and ex ΔCAR control NK-92 cells. Nonetheless, NK-92 cells modified with the full-length CARs still showed significantly enhanced killing against pHNSCC cells compared to unmodified NK-92 cells after 48 h co-culture (Figure 5A, Patient #16). Simultaneously, we analyzed target antigen expression on the remaining pHNSCC cells 24 h after co-culture to ensure adequate number of events for flow cytometric analysis. For all three patient samples, the HER1^+^/CD44v6^+^ cell population decreased while CD44v6^+^ pHNSCC cells expanded surprisingly after 24 h co-cultures with full-length or internally truncated CAR-NK-92 cells compared to unmodified, eGFP and ex ΔCAR control NK-92 cells. This expansion of CSC-like cells was especially apparent in samples #13 and #16 (Figure 5B). This expansion effect of internally truncated CAR-NK-92 cells is likely due to enhanced immune synaptic formation due to the anti-HER1 scFv expressed on the NK-92 cell surface. 

Incucyte analysis was performed to examine cytotoxicity and rate of apoptosis during co-culture of pHNSCC samples #14 (Figure 5C) and #16 (Figure 5D) with modified NK-92 cells. Cells from both pHNSCC patient samples were rapidly killed and showed an increased rate of apoptosis within the first 10 h of co-culture with both full-length CAR-NK-92 cells, similarly observed above for SCC-4 and SCC-25 cells. This indicates a rapid killing kinetic of the CAR-NK-92 cells against primary tumor cells even at a low E:T ratio. As previously seen in SCC-4 and SCC-25 cells, 12D3 CH3 and 2F8 CH3 CAR-NK-92 cells eliminated mCherry^+^ pHNSCC #16 cells nearly completely within 12 h. Furthermore, full-length CAR-NK-92 cells clustered around the few remaining pHNSCC #16 cells, which also displayed a visible increase in apoptotic cells as indicated by the higher Annexin V^+^ (blue) signal within these clusters (Figure 5E). Additionally, IFNγ secretion was also significantly upregulated by 12D3 CH3 CAR-NK-92 cells to during co-culture with pHNSCC #16. For 2F8 CH3 CAR-NK-92, this effect was also apparent, but not significantly different from CAR-NK-92 cells alone (Figure 5F). The MFI of CD107a was also significantly upregulated two-to-three-fold on both full-length CAR-NK-92 cell variants upon 4 h co-culture with pHNSCC samples #14, #15 and #16 (Figure 5G) compared to unmodified NK-92 monocultures.

### 3.6. Anti-HER1 CAR-NK-92 Treatment Resulted in Faster Spheroid Disruption

**Figure 6:**
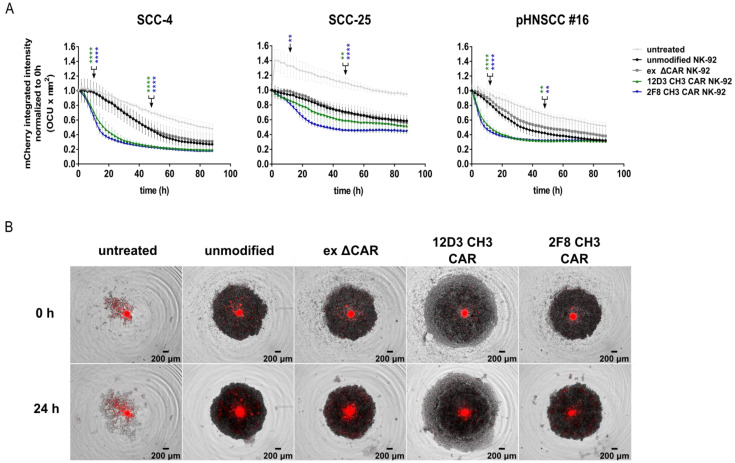
Efficient 3D spheroid killing upon co-culture with full-length 12D3 CH3 and 2F8 CH3 CAR-NK-92 cells. (**A**) Incucyte-based cytotoxicity assay using the 3D module and SCC-4 cell, SCC-25 cell and pHNSCC #16 derived spheroids. 5:1 E:T ratio was used for all target cell lines. (**B**) Automated live-cell imaging using Incucyte to detect pHNSCC #16 spheroids (red: mCherry^+^ pHNSCC #16 cells). E:T = effector:target cell ratio. Mean with standard deviation shown. Statistical analysis was performed using two-way ANOVA with Bonferroni’s post-hoc test for comparison between full-length CAR-NK-92 cells and unmodified NK-92 cells at 12 h and 48 h. n = 6 (two separate transductions of NK-92 used, each assessed in triplicates). ** = *p* < 0.01, **** = *p* < 0.0001.

To mimic the 3D nature of tumors, we generated spheroids of mCherry^+^ SCC-4, SCC-25 and pHNSCC #16 cells, for all of which we observed a similarly rapid decline in mCherry signal intensity within 12 h of co-culture with full-length CAR-NK-92 cells compared to untreated spheroids, or co-cultures with unmodified or ex ΔCAR-NK-92 control cells, as for the 2D cultures (Figure 6A,B). 

## 4. Discussion

HNSCC is a genetically complex, highly aggressive and relapse-prone form of cancer, requiring a multidimensional interplay of treatment regimens, in which immunotherapy has proven to play a pivotal role. In this study, we aimed to expand the immunotherapeutic tool kit to CAR-NK cell approaches against HNSCC. We present a functional CAR-NK-92 cell strategy targeting HER1 that was successfully tested against SCC-4 and SCC-25 as well as EpCAM sorted and expanded pHNSCC cells in 2D and 3D killing assays. 

To develop representative in vitro models of HNSCC, single tumor cells were isolated from inner tumor masses and characterized. High percentages of CD45^+^ immune cells were observed in 6 out of 9 samples, supporting HNSCC as one of the most infiltrated tumors [21]. Our flow cytometry and IHC data also support previous single-cell transcriptomic analysis, which identified 40% malignant and 60% non-malignant cells in 18 HNSCC samples [22]. Further investigation of the non-malignant population showed the presence of T cells, B cells, macrophages, monocytes, endothelial cells and fibroblasts. The composition of these non-malignant cell populations varied depending on tumor type, location, HPV status and genetic aberrations. Furthermore, HNSCC tumors were found to have one of the highest numbers of infiltrated, cytotoxic NK cells present, which correlated with improved overall survival due to the immune cell-mediated anti-tumor activity [21]. However, T cell populations also displayed exhaustion markers, which varied strongly among patient samples, suggesting an immunosuppressive TME, which may limit efficacy of immunotherapeutic approaches [22]. In this study, we did not characterize the immune cells to allow further conclusions. Generally, a high presence of immune cells, specifically NK cells and macrophages, correlates with better prognosis and validates investigations of immunotherapeutic approaches for these tumors [23].

Expression patterns of HER1, CD44v6 and CD133 on freshly isolated pHNSCC samples showed HER1 had the highest expression of the three, reinforcing our choice as a therapeutic target and supporting previous literature showing over 80% HER1 expression. CD133 displayed the lowest expression, as expected from previous work that described CD133 as a putative cancer stem cell marker and CD133^+^ cells as a rather rare subpopulation [24]. The CD45^−^ population consisted of a large portion of EpCAM^+^ cells and as epithelial cells are the cell-of-origin for this type of tumor, we sorted freshly isolated pHNSCC samples for EpCAM as described by others and expanded the samples in an epithelial cell-specific medium [20]. This procedure helped avoid fibroblast overgrowth in cell culture flasks without the need to use the partial trypsinization method over weeks to months with a success rate below 25% [20,25]. After EpCAM^+^ sorting, HER1 expression reached over 50% in 5 out of 7 freshly isolated patient samples. Overall, this allowed us to create pHNSCC-derived cell lines, which persisted in culture for months with consistent epithelial morphology (Supplementary Figure S4), allowing freeze/thaw cycles and transduction with lentiviral vectors (e.g., for mCherry expression). This isolation and culture strategy generated a sufficient number of cells from primary material, which enabled us to conduct different experiments using the same pHNSCC cell line.

To further characterize EpCAM sorted and expanded pHNSCC samples, we conducted OGM, which alongside SCC-4 and SCC-25 cell lines, displayed the highly heterogeneous background and genetic complexity of the disease. SCC-4 cells showed the highest number of genomic aberrations, which may explain the higher E:T ratio needed to effectively kill these cells compared to SCC-25 and pHNSCC cells and suggests that SCC-4 cells may reflect a rather resistant model of HNSCC for this type of therapy. However, all cell lines also shared a high-fold amplification of chromosomal region 11q13, which encodes genes involved in cell cycle control that are regarded as oncogenic drivers, such as *CCND1, FADD* and *CTTN* [18]. This also supports the finding that pHNSCC samples that harbor gene amplification of chromosomal region 11q13, which serves as a marker for poor prognosis, were more likely to form stable cell lines [26]. 

To establish a functional anti-HER1 CAR-NK cell strategy, a triple parameter reporter (TPR) cell line was used to test our CAR construct for tonic signaling, and showed signaling for 7D6 CH3 in the absence of target antigen, which can lead to exhaustion in CAR-T cells (Supplementary Figure S3) [27,28]. From the remaining CAR candidates, we selected 12D3 CH3 and 2F8 CH3 as the best candidates to compare two different scFvs in all further experiments. These two most promising full-length CAR candidates outperformed all controls in enhanced cytotoxic killing against SCC-4, SCC-25 and both pHNSCC cell lines. Additionally, both full-length CAR-NK-92 cells demonstrated enhanced apoptosis induction in all target cells as well as increased CD107a expression, indicating degranulation of granzyme and perforins as mechanisms of apoptosis induction [29,30]. Another main function of NK cells is the recruitment and activation of other immune cells, such as monocytes, T cells and B cells to elicit an adaptive immune response, which is induced by enhanced secretion of IFNγ. These two full-length CAR-NK-92 cells exhibited IFNγ secretion upon co-culture with target cells SCC-4, SCC-25 and pHNSCC cell lines. Furthermore, IFNγ has also been shown to directly enhance NK cell-mediated induction of cancer cell death by apoptosis and cytolysis [31,32]. We also found enhanced cytotoxic killing of 3D spheroids using SCC-4, SCC-25 and pHNSCC cells in co-culture with both full-length CAR-NK-92 cells, confirming results from the 2D killing assays. For 3D models, higher E:T ratios were used for SCC-25 and pHNSCC cells to see an enhanced spheroid killing in co-cultures with full-length CAR-NK-92 cells, underlining more complex cellular interactions and most likely limited access to target antigens compared to 2D models, which results in increased treatment resistance as previously demonstrated [33].

Since strategies targeting single antigens can lead to resistance development, such approaches require close observation of tumor progression and resistance [34,35]. Therefore, we examined antigen expression profiles of remaining pHNSCC cells and found a strong reduction of HER1^+^ and CD44v6^+^HER1^+^ target cells, as expected, with increased CD44v6^+^ cells present after 24 h co-culture with full-length CAR-NK-92 cells. This may indicate expansion of clonal subsets as a response to CAR treatment, specifically the potential of cells that may resemble cancer stem cells to spread and repopulate. Antibodies against CD44v6 were shown to prevent such outgrowth in rat models of metastatic cancer [36]. Treatment resistance is another concern, as patients treated with cetuximab developed resistance to cetuximab as well as decreased responses to PD-1 checkpoint inhibitors in subsequent treatments [37]. It is well-known that other receptors of the ErbB family or downstream signaling molecules can compensate for the inhibition of one receptor [38], further supporting the idea of dual-targeted CAR-NK cell approaches. A previous study demonstrated enhanced and specific killing of primary HNSCC cells using CAR-T cells targeting CD44v6 [39]. A CAR strategy that targets both HER1 and CD44v6 using human T and NK cells may help to further eliminate putative cancer stem cell populations more completely, with the aim to reduce tumor escape and relapse incidences. 

Another major drawback of adoptive cell therapy approaches is the upregulation of PD-L1 on target cells, which we also observed for SCC-4, SCC-25 and pHNSCC cells upon co-culture with unmodified and modified NK-92 cells (Supplementary Figure S5C) [40]. CAR-NK cell therapy may hence benefit from the combination of checkpoint inhibitors to prevent loss of immune cell functionality and aid in overcoming resistance in the immunosuppressive TME of HNSCC, which remains a challenge for any CAR therapeutic approach [41]. Further experiments are therefore required to assess performance of our anti-HER1 CAR-NK-92-cell strategy in a model that better recapitulates an immunosuppressive TME [42,43,44]. While further research is inevitable, HNSCC remains a disease that requires multimodal treatment regimens. Therefore, it is mandatory that new treatment strategies can be implemented into and further advance existing therapeutic strategies. Multiple studies have already shown beneficial outcomes of combined (CAR)-NK cell therapies and radiation or chemotherapy in other cancer models, making it a promising new therapeutic approach for HNSCC [45,46,47,48]. 

## 5. Conclusions

In the present study, CAR-NK-92 cells that target HER1 were generated and showed enhanced killing activity of SCC cell lines and established patient-derived pHNSCC cells in 2D and 3D co-culture models. Furthermore, improved IFNγ secretion and upregulated expression of CD107a were observed during co-culture with anti-HER1 CAR-NK-92 cells. However, characterization of remaining pHNSCC cells after co-culture with anti-HER1 CAR-NK-92 cells demonstrated an expansion of CD44v6^+^ cells, which may be indicative of a CSC-like phenotype. Together with the upregulated PD-L1 expression on pHNSCC cells after co-culture with anti-HER1 CAR-NK-92 cells, this suggests CAR-based approaches targeting a single tumor-associated antigen may not be suitable for monotherapy, and hence, may not be able to prevent high relapse rates and treatment resistance. Therefore, combined immunotherapeutic approaches that target multiple tumor-associated antigens as well as the immunosuppressive TME are essential to improve treatment responses and survival rates for HNSCC.

## Figures and Tables

**Table 1:** Primary HNSCC patient samples analyzed.

Sample	Region	HPV Status	Tumor Occurrence	Treatment Post Biopsy
#1	Tongue	negative	primary	primary RCTx
#2	Tonsil right	negative	primary	primary RCTx
#3	Parapharyngeal	/	primary	primary RCTx
#4	Nose skin	negative	primary	surgery, no RCTx
#5	Tonsil left	positive	primary	surgery, no RCTx
#6	Soft palate	negative	primary	primary RCTx
#7	Oropharynx right	negative	primary	primary RCTx
#8	Larynx	/	primary	/
#9	Tongue basis	positive	primary	primary RCTx
#10	Hypolarynx	negative	primary	primary RCTx
#11	Larynx	negative	secondary	surgery, no RCTx
#12	Uvula	positive	primary	none
#13	Uvula	negative	primary	primary RCTx
#14	Tonsils	negative	primary	primary RCTx
#15	Tonsil	negative	primary	primary RCTx
#16	Tongue	negative	secondary	Surgery, no RCTx

RCTx = radio- and chemotherapy, / = no information available.

## Data Availability

The data presented in this study are available in the article and Supplementary Material.

## Supplementary Materials

The following supporting information can be downloaded at: https://www.mdpi.com/article/10.3390/cancers15123169/s1. Supplementary Figure S1: Characterization of SCC-4 and SCC-25 cell lines; Supplementary Figure S2. Enhanced IFNγ secretion of anti-HER1 CAR-NK-92 cells using E:T ratio 1:1 and 10:1. Supplementary Figure S3. Tonic signaling of anti-HER1 CAR-TPR cell line; Supplementary Figure S4. EpCAM expression on target cell lines; Supplementary Figure S5. Upregulation of PD-L1 on target cells upon NK-92 co-culture; Supplementary Figure S6. Immunohistochemistry staining of patient #16; Supplementary Figure S7. Immunohistochemistry staining of patient #15. Supplementary methods include Flow cytometry: surface marker expression and Immunohistochemistry staining and analysis (including Supplementary Table S1: Antibodies used in immunohistochemistry). File S1: Original Images of Western Blot.
