# Supplementary material for: CAR-NK Cells Targeting HER1 (EGFR) Show Efficient Anti-Tumor Activity against Head and Neck Squamous Cell Carcinoma (HNSCC)
Source: Cancers (Basel). 2023 Jun 13;15(12):3169. doi: 10.3390/cancers15123169 (PMC10296665; doi:10.3390/cancers15123169)
Supplement: Supplementary file 1 [file cancers-15-03169-s001.zip › cancers-2414510-supplementary Figures and Tables.pdf]

1. Supplementary figures

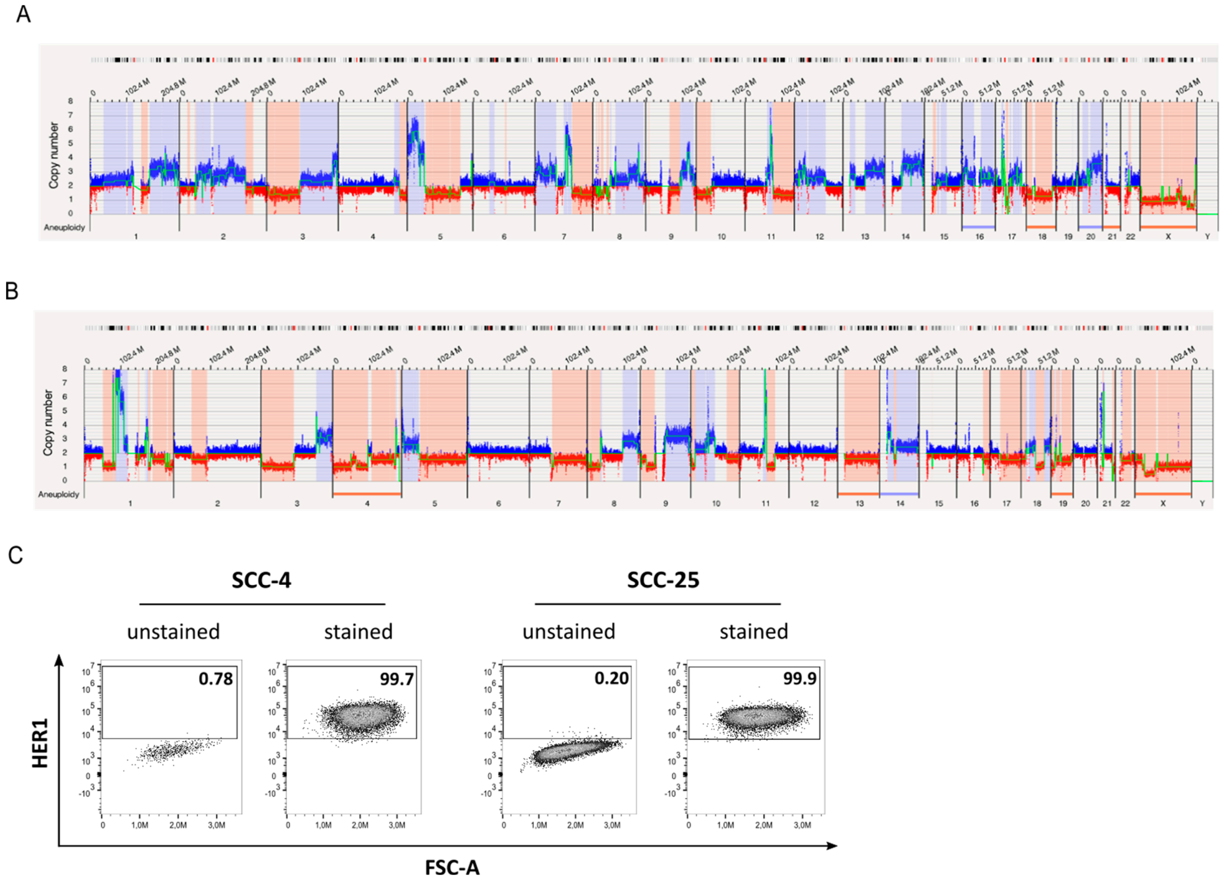

Supplementary Figure S1. Characterization of SCC-4 and SCC-25 cell lines. Optical genome mapping of **(A)** SCC-4 cells and **(B)** SCC-25 cells. Depicted copy number gains (●) and losses (●) for each chromosome (>500 kbp). **(C)** HER1/EGFR expression on SCC-4 and SCC-25 cell lines assessed via flow cytometry.

A

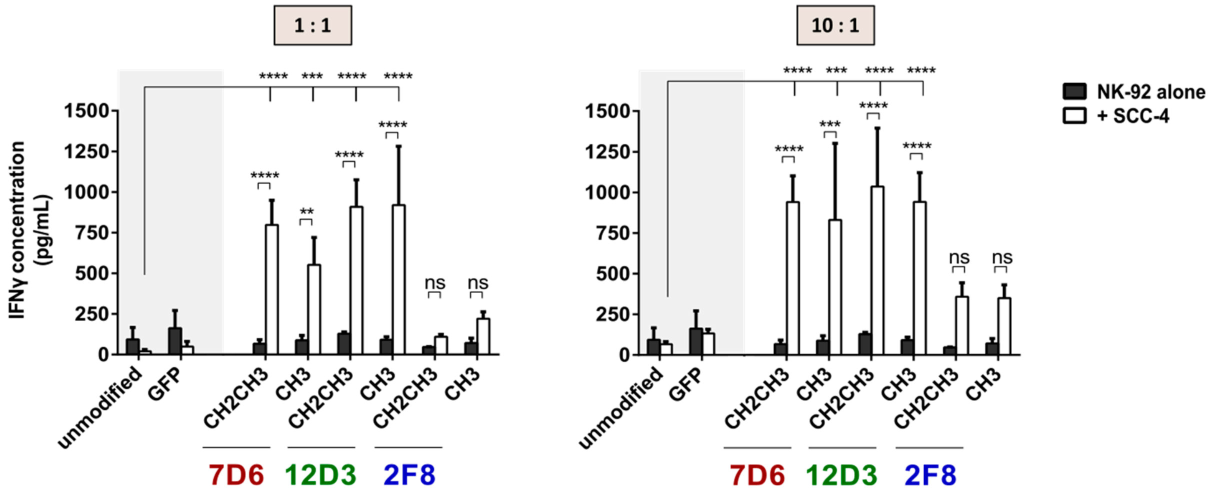

Supplementary Figure S2. Enhanced IFNγ secretion of anti-HER1 CAR-NK-92 cell variants using E:T ratios of 1:1 and 10:1. (A) Supernatants from 48 h co-cultures with and without SCC-4 cells at E:T of 1:1 and 10:1 were collected and analyzed for IFNγ secretion via ELISA. E:T = effector:target cell ratio. Mean with standard deviation shown. Statistical analysis was performed using two-way ANOVA with Bonferroni's post-hoc test for comparison to unmodified NK-92 cells within the same time points (e.g. 24 h to 24 h), n = 3.

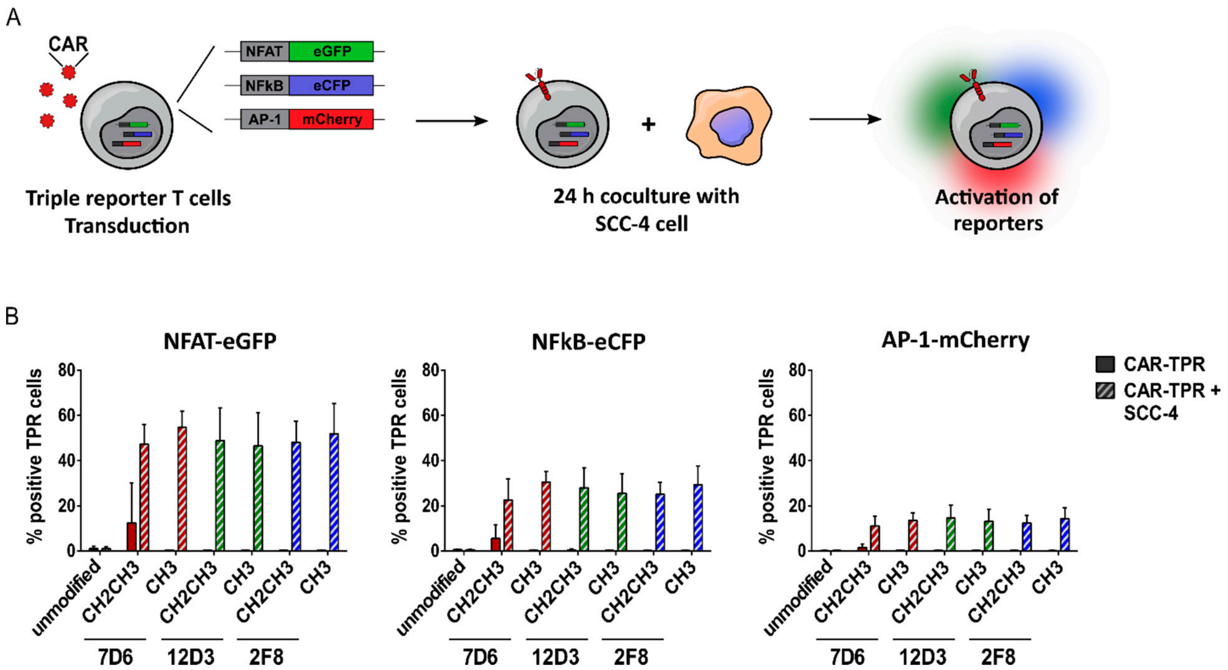

Supplementary Figure S3. Tonic signaling of anti-HER1 CAR-TPR cell line. (A) Experimental design of triple parameter Jurkat cells (NFAT-eGFP, NFkB-eCFP, AP1-mCherry) modified with six anti-HER1 CAR variants (E:T ratio = 10:1). Activation of reporters analysed by flow cytometry. **(B)** Activation of reporters of CAR-TRP cells in monoculture or co-culture with SCC-4 cells. E:T = effector:target cell ratio. Mean with standard deviation shown, n = 3-5.

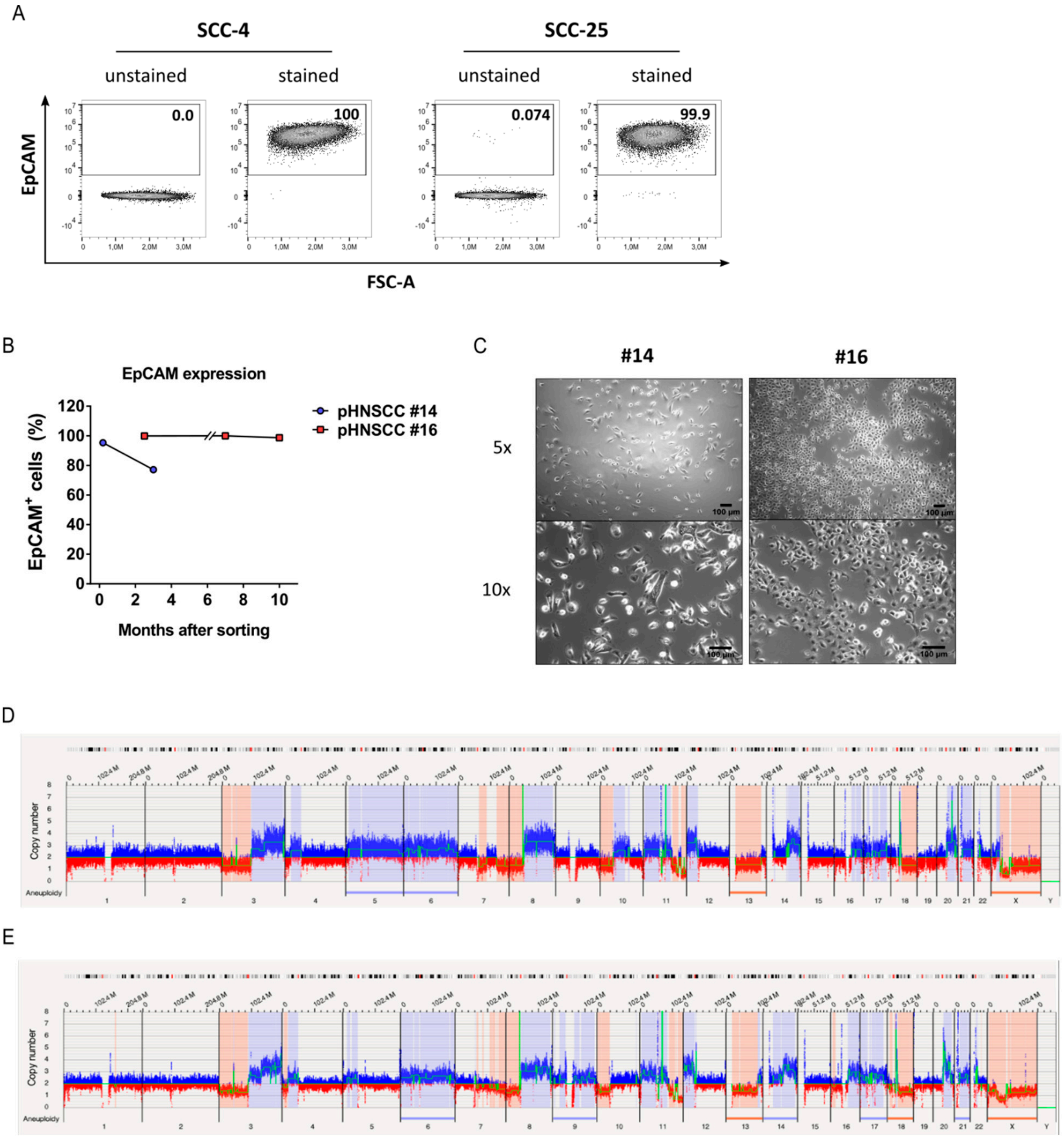

Supplementary Figure S4. EpCAM expression on target cell lines. (A) EpCAM expression on SCC-4 and SCC-25 cells. **(B)** EpCAM expression on EpCAM-sorted and expanded pHNSCC cells #14 and #16 over time. Timeline disruption (//) indicates freezing/thawing cycle. **(C)** Morphology of EpCAM sorted pHNSCC cells #14 and #16 in 5x and 10x magnification (Scale bar indicates 100 µm). Optical genome mapping of EpCAM sorted pHNSCC samples **(D)** #14 and **(E)** #16. Depicted copy number gains (●) and losses (●) for each chromosome (>500 kbp).

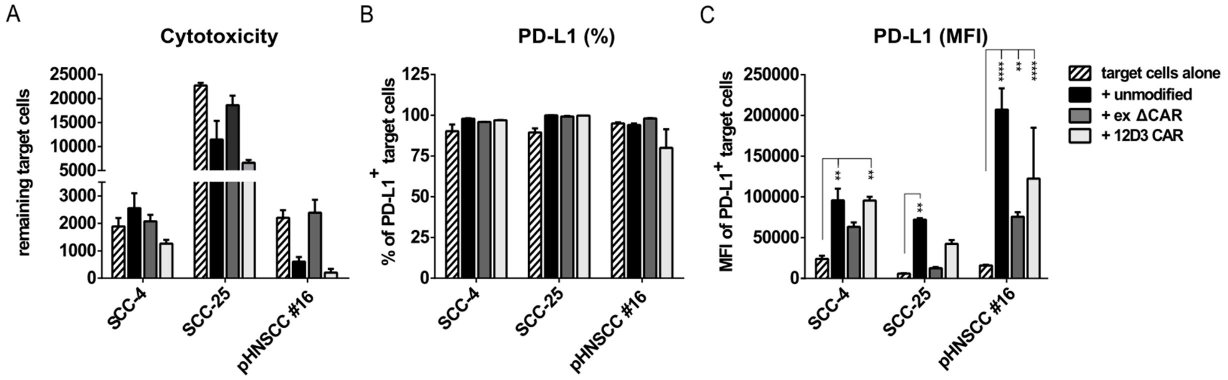

Supplementary Figure S5. Upregulation of PD-L1 on target cells upon NK-92 co-culture. (A) Percentage of remaining target cells 24 h after monoculture (target cells alone) or co-culture with unmodified, ex ΔCAR or full length anti-HER1 12D3 CH3 CAR-NK-92 cells (SCC-4 cells = 1:1 E:T ratio; SCC-25 and pHNSCC #16 = 0.5:1 E:T ratio). Expression of PD-L1 on remaining at target cells after 24 h monoculture or co-culture in **(B)** percentages and **(C)** MFI. E:T = effector:target cell ratio. Mean with standard deviation shown. Statistical analysis performed using two-way ANOVA with Bonferroni's post-hoc test, n = 3.

A

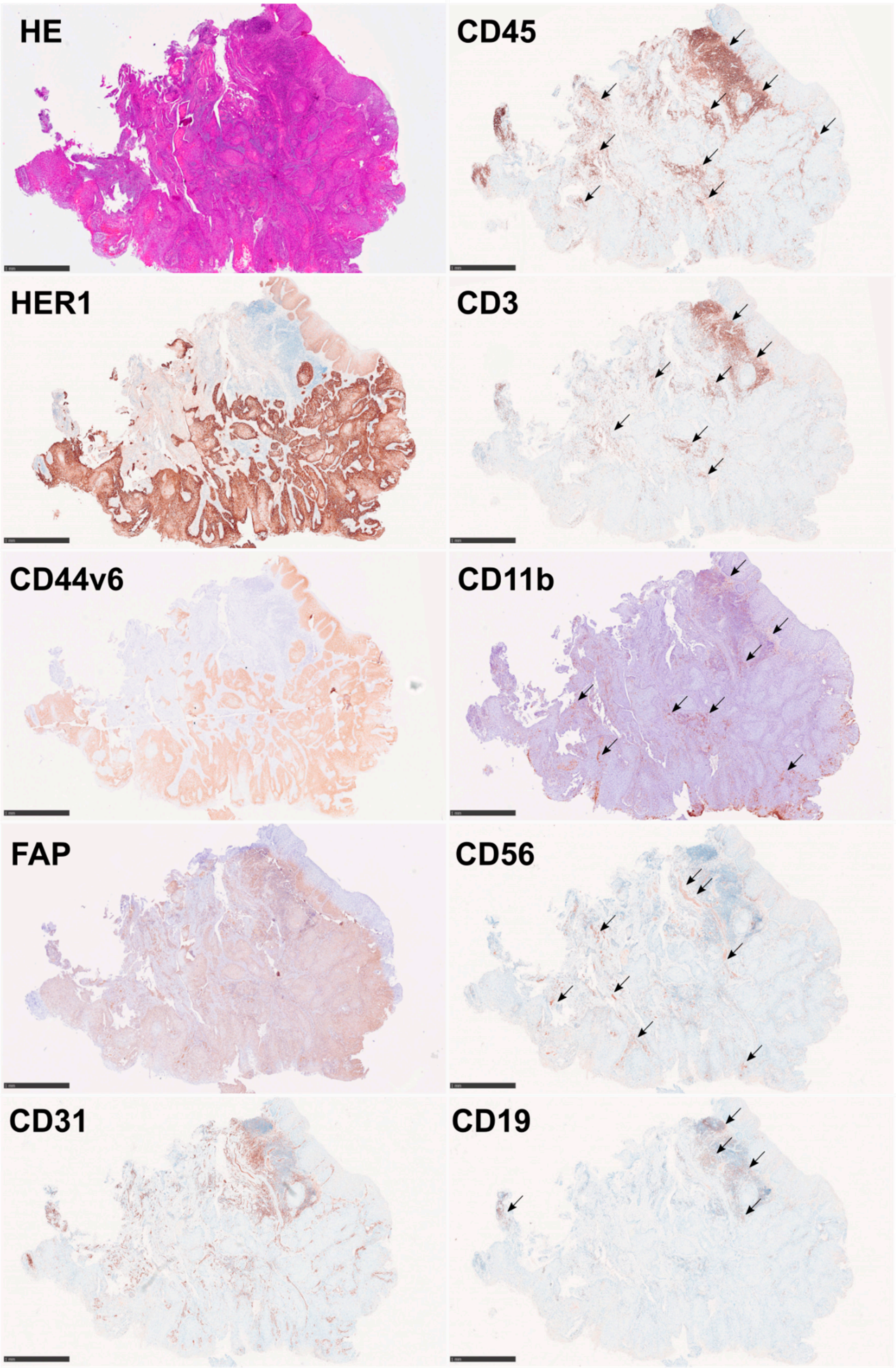

Supplementary Figure S6. Immunohistochemistry staining of patient #16. (A) Different immunohistochemistry stainings were performed on paraffin sections. HE staining showed pink nodules confirming squamous cell carcinoma, which also demonstrated strong HER1 expression. HER1 expression was co-localized with CD44v6 expression. $CD45^+$ cells mainly localized to regions lacking $HER1^+$ and $CD44v6^+$ cells. $CD45^+$ regions mainly contained $CD3^+$ cells and $CD11b^+$ cells, and to a lower extent $CD56^+$ cells and $CD19^+$ cells. Immunohistochemistry (IHC) confirms presence of FAP^+ and $CD31^+$ cells. HE = hematoxylin and eosin, IHC = immunohistochemistry.

A

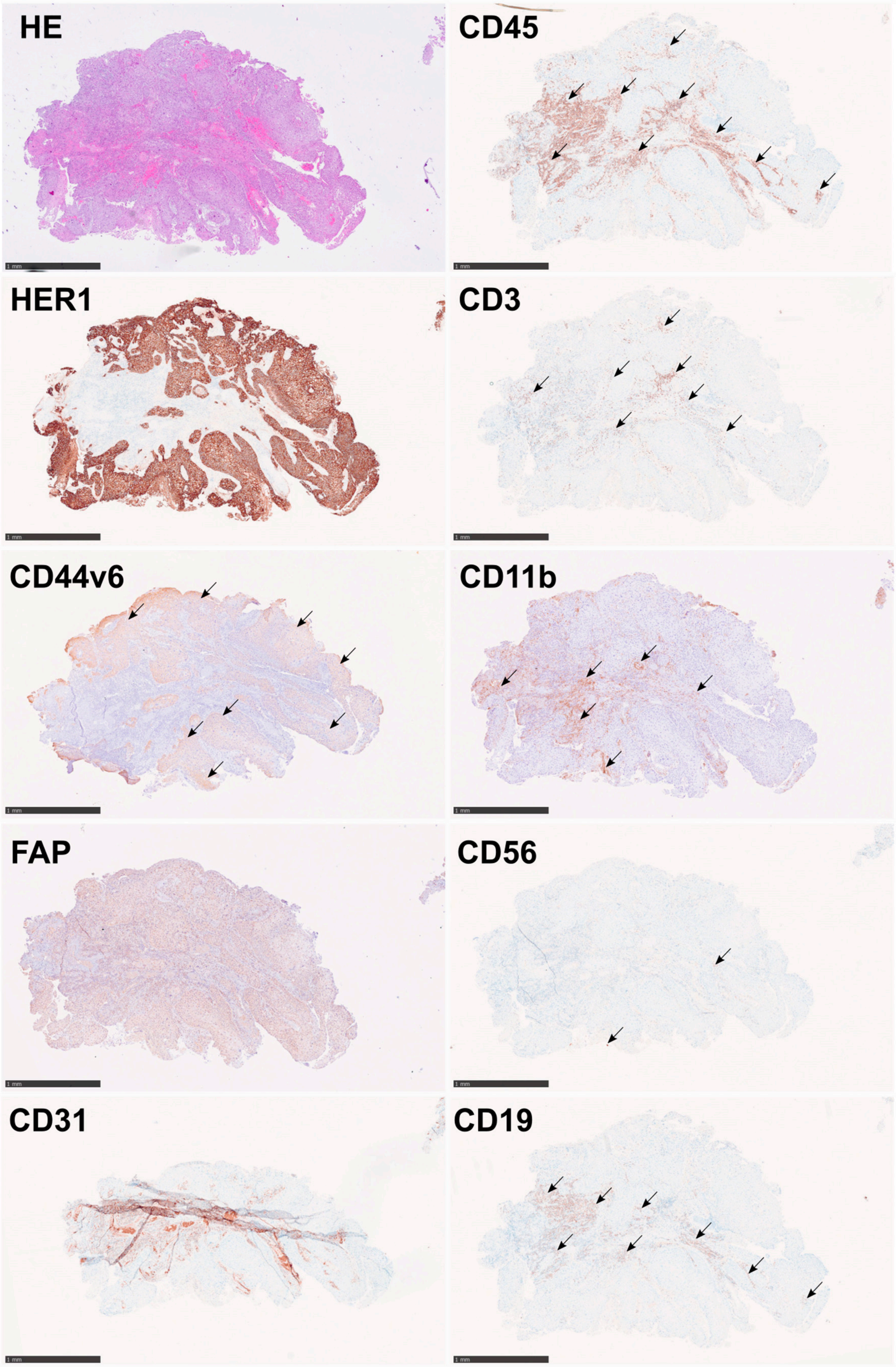

Supplementary Figure S7. Immunohistochemistry staining of patient #15. (A) Different immunohistochemistry stainings were performed on paraffin sections. HE staining showed pink nodules confirming squamous cell carcinoma, which also demonstrated strong HER1 expression. HER1 expression was co-localized with CD44v6 expression. $CD45^+$ cells mainly localized to regions lacking $HER1^+$ and $CD44v6^+$ cells. $CD45^+$ regions mainly contained $CD3^+$ cells and $CD11b^+$ cells, and to a lower extent $CD56^+$ cells and $CD19^+$ cells. Immunohistochemistry (IHC) confirms presence of FAP^+ and $CD31^+$ cells. HE = hematoxylin and eosin, IHC = immunohistochemistry.

2. Supplementary materials and methods

2.1. Flow cytometry: surface marker expression

The following anti-human antibodies were used for flow cytometry analysis using CytoFLEX S (Beckman Coulter, Brea, CA, USA): DYKDDDDK-tag-APC (flag-tag) (BioLegend, San Diego, CA, USA), HER1/EGFR-APC (BioLegend, San Diego, CA, USA), HER1/EGFR-BV605 (BioLegend, San Diego, CA, USA), CD44v6-PE (R&D Systems, MN, USA), CD133-APC (BioLegend, San Diego, CA, USA), CD45-BV650 (BD Biosciences, Franklin Lakes, NJ, USA), CD56-PC7 (BD Biosciences, Franklin Lakes, NJ, USA), CD3-BV785 (BioLegend, San Diego, CA, USA), CD19-FITC (Miltenyi Biotec, Bergisch Gladbach, Germany), CD11b-PE (Miltenyi Biotec, Bergisch Gladbach, Germany), EpCAM-PE (BioLegend, San Diego, CA, USA), FAB-FITC (BioLegend, San Diego, CA, USA), CD31-APC (Miltenyi Biotec, Bergisch Gladbach, Germany), CD140a-PerCP-Cy5.5 (Miltenyi Biotec, Bergisch Gladbach, Germany). Analysis was performed using FlowJo10 (BD Biosciences, Franklin Lakes, NJ, USA).

2.2. Immunohistochemistry staining and analysis

Stainings were performed using the antibodies listed in supplementary table 1.

Supplementary Table S1. Antibodies used for immunohistochemistry

Target of antibody	Product name	Host species	Pretreatment	Dilution	Manufacturer
**CD11b**	Recombinant Anti-CD11b antibody [EPR1344] (ab133357)	Rabbit	Tris-EDTA buffer	1:2000	Abcam
**CD19**	CD19 antibody (NCL-L-CD19-163)	Mouse	Tris-EDTA buffer	1:50	Leica
**CD3**	CONFIRM anti-CD3 (2GV6) Rabbit Monoclonal Primary Antibody	Rabbit	Tris-EDTA buffer	RTU	Roche
**CD31**	CD31 antibody (JC70)	Mouse	Tris-EDTA buffer	RTU	Roche
**CD44v6**	CD44var (v6) Monoclonal Antibody (VFF-18) (BMS125)	Mouse	Tris-EDTA buffer	1:200	eBioScience

**CD45**	CD45 (LCA) (2B11 & PD7/26)	Mouse	Tris-EDTA buffer	RTU	Roche
**CD56**	CD56 antibody (MRQ-42)	Rabbit	Tris-EDTA buffer	RTU	Roche
**EGFR**	CONFIRM anti-Epidermal Growth Factor Receptor (3C6) Primary Antibody	Mouse	Protease 1 pretreatment (8 minutes)	RTU	Roche
**EpCAM**	Recombinant Anti-EpCAM antibody [VU-1D9] (ab187372)	Mouse	Protease 1 pretreatment (4 minutes)	1:100	Leica
**FAP**	Anti-Fibroblast activation protein, alpha antibody (ab53066)	Rabbit	Tris-EDTA buffer	1:100	Abcam
**PD-1**	PD-1 (NAT105) Mouse Monoclonal Antibody	Mouse	Tris-EDTA buffer	1:400	Cell Marque

CD11b/19/3/31/44v6/45/56, cluster of differentiation 11b/19/3/31/44v6/45/56; EGFR, epidermal growth factor receptor; EpCAM, epithelial cell adhesion molecule; FAP, fibroblast activated protein; PD-1, programmed cell death 1. EDTA, ethylenediamine tetraacetic acid; RTU, ready to use concentration provided by manufacturer.
