# Supplementary figures and images for: CAR-NK Cells Targeting HER1 (EGFR) Show Efficient Anti-Tumor Activity against Head and Neck Squamous Cell Carcinoma (HNSCC)
Source: Cancers (Basel). 2023 Jun 13;15(12):3169. doi: 10.3390/cancers15123169 (PMC10296665; doi:10.3390/cancers15123169)

Original Images of Western Blot:

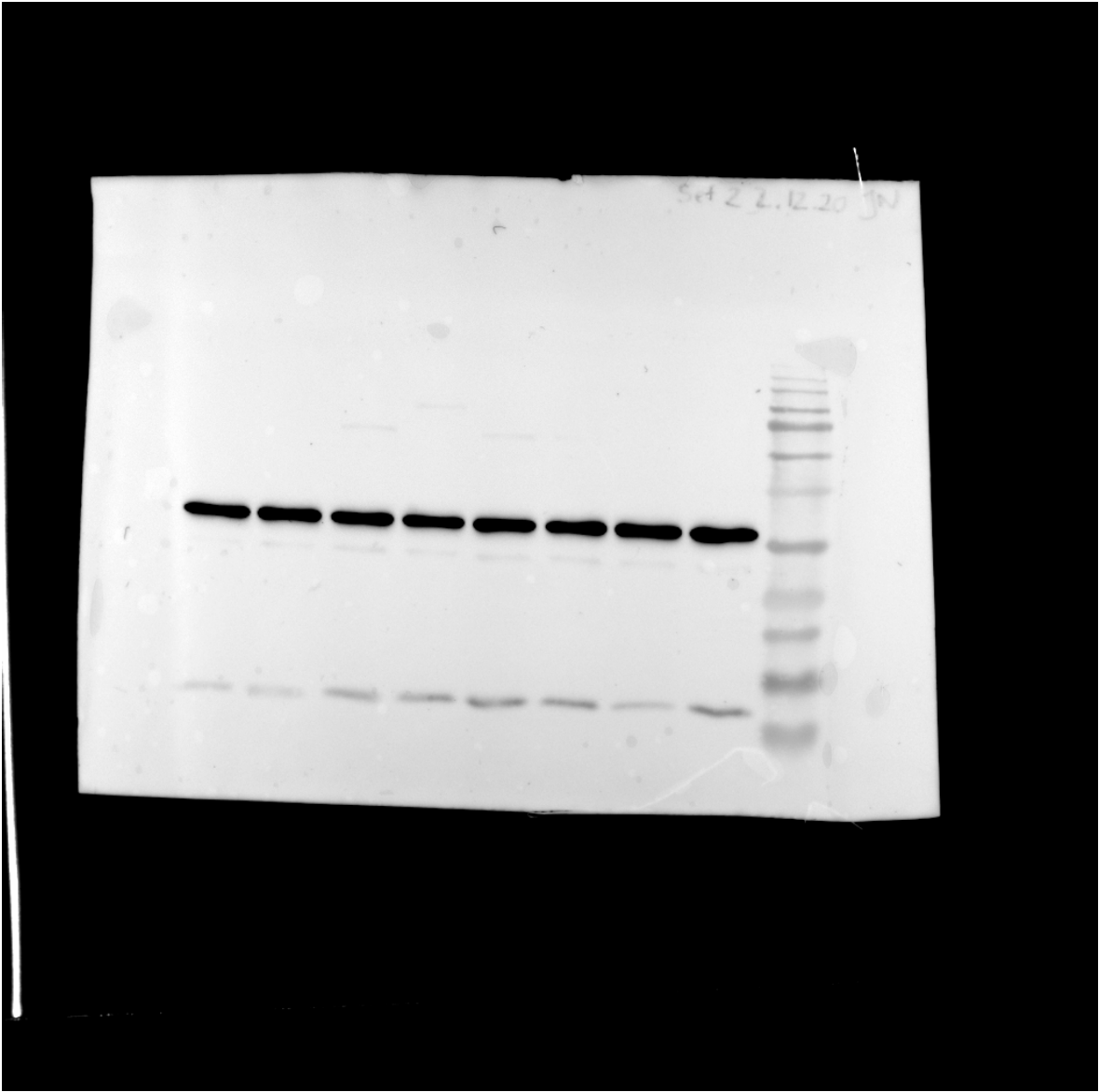

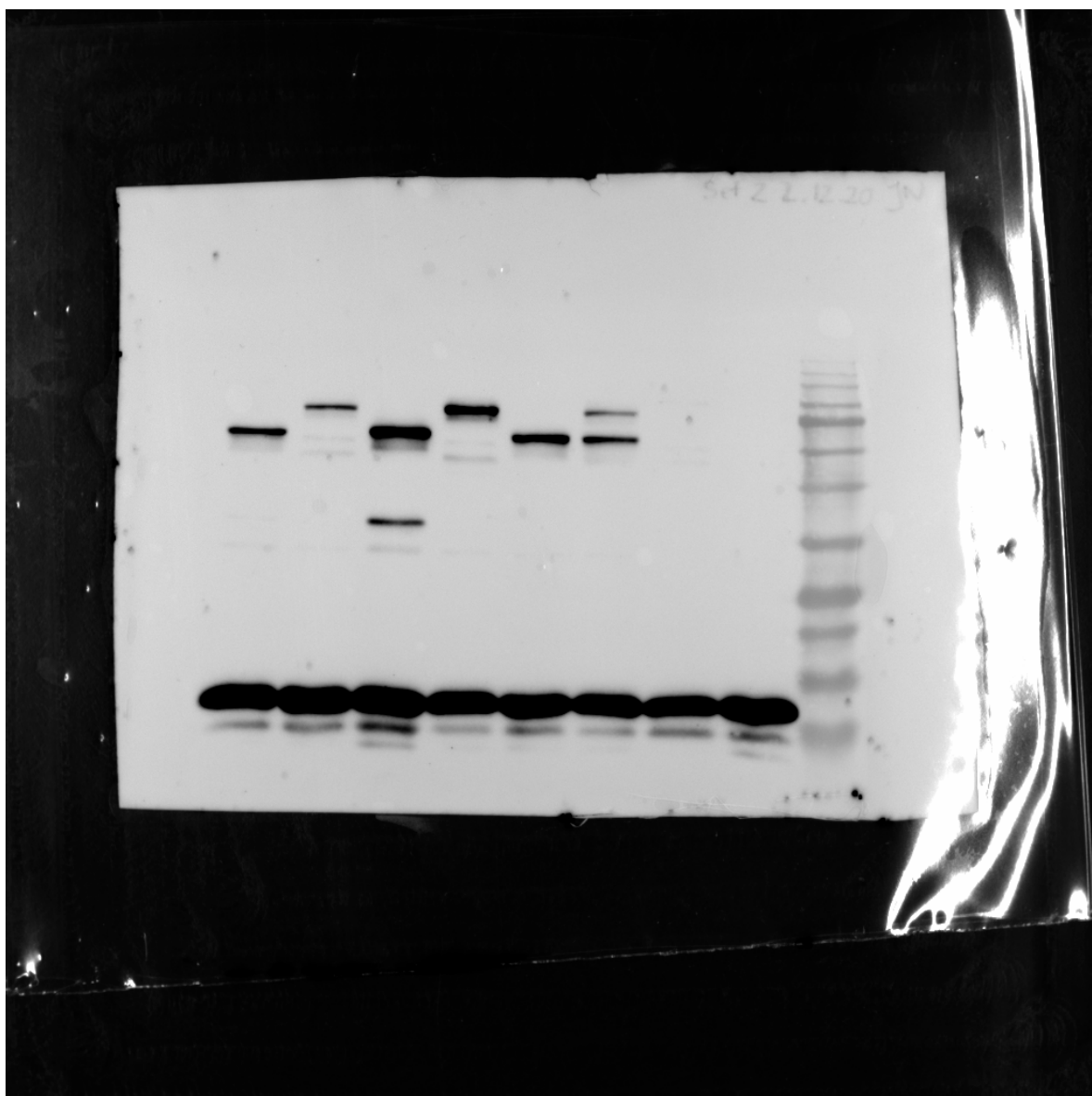

Supplement: Supplementary file 1 [file cancers-15-03169-s001.zip › cancers-2414510-Supplementary File S1.pdf]
